# Systematic review and meta-analysis of the intervention effect of curcumin on rodent models of myocardial infarction

**DOI:** 10.3389/fphar.2022.999386

**Published:** 2022-10-18

**Authors:** Bing-Yao Pang, Ya-Hong Wang, Xing-Wang Ji, Yan Leng, Hou-Bo Deng, Li-Hong Jiang

**Affiliations:** ^1^ College of Traditional Chinese Medicine, Changchun University of Traditional Chinese Medicine, Changchun, China; ^2^ Department of Hepatology, Affiliated Hospital of Changchun University of Traditional Chinese Medicine, Changchun, China; ^3^ Department of Emergency, The First Clinical Hospital of Jilin Academy of Traditional Chinese Medicine, Changchun, China; ^4^ Department of Cardiovascular Medicine, Affiliated Hospital of Changchun University of Traditional Chinese Medicine, Changchun, China

**Keywords:** curcumin, myocardial infarction, model, systematic evaluation, meta-analysis

## Abstract

**Objective:** This study aimed to evaluate the intervention effect of curcumin in myocardial infarction rodent models.

**Methods:** A systematic retrieval of relevant studies on curcumin intervention in rats or mice myocardial infarction models was conducted, and the data were extracted. The outcome indicators included biochemical blood indicators, such as creatine kinase (CK), creatine kinase isoenzyme (CK-MB), malondialdehyde (MDA), lactate dehydrogenase (LDH) and superoxide dismutase (SOD), as well as cardiac tissue structure indicators, such as left ventricular weight to body weight ratio (LVW/BW), apoptosis index, left ventricular end-diastolic dimension (LVEDD), left ventricular end-systolic diameter (LVESD), and myocardial infarction area, and hemodynamic indexes, such as systolic blood pressure (SBP), diastolic blood pressure (DBP), left ventricular end-diastolic pressure (LVEDP), left ventricular ejection fraction (LVEF), left ventricular fractional shortening (LVFS), maximum rate of left ventricular pressure rise (+dp/dtmax), and maximum rate of left ventricular pressure decline (–dp/dtmax). These results were then analyzed by meta-analysis. Studies were evaluated for methodological quality using the syrcle’s bias risk tool.

**Results:** A total of 24 studies were included in the meta-analysis. The quality assessment of included studies revealed that the evidence was low quality and none of studies was judged as having a low risk of bias across all domains. The results revealed that curcumin could reduce CK-MB, CK, LDH, and MDA levels. They also revealed that it could lower SBP, DBP, LVEDP, LVW/BW, apoptosis index, LVEDD, LVESD, and myocardial infarction area and increase LVEF, LVFS, +dp/dtmax, and–dp/dtmax. However, it had no significant impact on the heart rate and the levels of SOD in the models.

**Conclusion:** Curcumin alleviates myocardial injury and oxidative stress in myocardial infarction rodent models in terms of blood biochemistry indicators, improves the diastolic and systolic capacity of the ventricle in terms of hemodynamic indexes, and reduces the necrosis and apoptosis of cardiomyocytes in terms of tissue structure. The methodological quality of the studies was low and additional research is warranted.

## Introduction

Myocardial infarction refers to the necrosis of any size of myocardial cells caused by myocardial ischemia (Sumitra et al., 2007). Myocardial infarction seriously endangers human health and has a very high incidence and mortality worldwide (Boarescu et al., 2019a). In the report on cardiovascular health and diseases in China 2021: an updated summary, the mortality rate of myocardial infarction in China in 2019 was 60.2/100000 in urban areas and 78.24/100000 in rural areas, about half of the mortality rate of all kinds of tumors. The number of discharges from acute myocardial infarction in China in 2019 was 1,057,600. The epidemiological study on myocardial infarction in Korea points out that the prevalency in adults over 30 years was estimated to range from 0.34% to 0.70% in 2020 (Kim et al., 2022). The mortality was 19.3 per 100000 populations In Denmark, from 1 January 2005, through 4 August 2021, there were 116481 inch acute myocardial informations in approximately 4.5 million Danes aged ≥18 years (Christensen et al., 2022). With the extensive implementation of thrombolysis, cardiac interventional surgery, and the development of drug treatment, the dying myocardium can be effectively saved, and the prognosis of patients with myocardial infarction has greatly improved in recent years. However, many patients do not receive revascularization in time for various reasons, resulting in irreversible death of the myocardium, ventricular remodeling, deterioration of cardiac function, and finally, heart failure, which seriously affects a patient’s quality of life (Boarescu et al., 2019b).

Curcumin is a phenolic compound extracted from the roots of plants in the Zingiberaceae family (Duan et al., 2012; Lestari and Indrayanto, 2014). In recent years, it has been shown to have a wide range of pharmacological effects. In terms of cardiovascular disease, it has also been proven to protect against myocardial ischemia-reperfusion injury (Li et al., 2020), and there are many studies on its role in animal models. To further clarify the mechanism of curcumin on myocardial infarction, this study carried out a meta-analysis to evaluate the intervention effects of curcumin on rat and mouse myocardial infarction models to provide a reference and evidence for the clinical applications of curcumin.

## Materials and methods

### Literature review

Related studies were retrieved from PubMed, Embase, Cochrane Library, Web of Science, CNKI, VIP, and Wanfang. The time range was from the establishment of the databases to 9 June 2022. The keywords used for retrieval included three parts: 1) curcumin, 2) myocardial infarction, 3) rats or mice, and the three parts were connected by “and.”

The inclusion criteria for the studies were as follows:

1) Research subject: a rat or mouse myocardial infarction model; the preparation method of the model had to be recognized.
2) Intervention measures: The drugs used had to be curcumin or curcumin preparations. The experimental group could not additionally use other drugs that may affect the heart; the control group did not use any drugs, or only used a placebo.
3) Outcome indicators: The indicators for evaluating drug effects had to be in digital form to ensure that the key indicators could be extracted or calculated directly or indirectly, including their mean and standard deviation.

The exclusion criteria were as follows:

1) The data for evaluation indicators was incomplete.
2) Information regarding meta-analysis, systematic evaluation, and correspondence was incomplete.
3) The experimental group or the control group involved the use of other drugs.
4) The study involved animals other than a rat or mouse.

Two authors extracted data independently. Any differences were resolved through discussion until a consensus was reached, or a third author was consulted to add a decisive input. The following pieces of information were extracted from qualifying studies: author, year of publication, drugs used, modeling method, number of models, mode of administration, dosage, detection indexes, detection index unit, and detection results (Cheng and Liu, 2005; Wang, 2008; Hong et al., 2009; Gao et al., 2011; Sunagawa et al., 2011; Kim et al., 2012; Kong, 2012; Sunagawa et al., 2012; Chen, 2014; Sunagawa et al., 2014; Chen et al., 2015; Gao et al., 2015; He et al., 2015; Liu et al., 2015; Xu and Yang, 2015; Geng, 2016; Gu et al., 2016; Liu, 2018; Boarescu et al., 2019a; Mehdi et al., 2019; Cui et al., 2020; Guo et al., 2021; HJ W et al., 2021; Shaoxi et al., 2021). Finally, the outcome indicators were summarized. When there were less than three articles on the outcome indicators, the outcome indicators were excluded from the study.

To assess the quality of the included studies, we used the SYstematic Review Center for Laboratory Animal Experimentation bias risk tool (syrcle’s bias risk tool) for animal studies (Musillo et al., 2021; Wang et al., 2021). Syrle’s bias risk tool is based on the Cochrane bias risk tool and adjusted for bias aspects that play a specific role in animal intervention studies. Two authors extracted data independently. Any differences were resolved through discussion until a consensus was reached, or a third author was consulted to add a decisive input.

### Statistical methods

Meta-analysis was conducted using RevMan 5.1 software. Quantitative data were expressed as mean ± standard deviation (±SD). Cochran’s Q test and the I^2^ test were used to evaluate the existence and severity of heterogeneity were. When both *p* < 0.1 and I^2^ > 50%, it was considered that there was heterogeneity, and the random-effects model was used for meta-analysis; otherwise, the fixed-effects model was used.

## Results

### Results of the literature review

**FIGURE 1:**
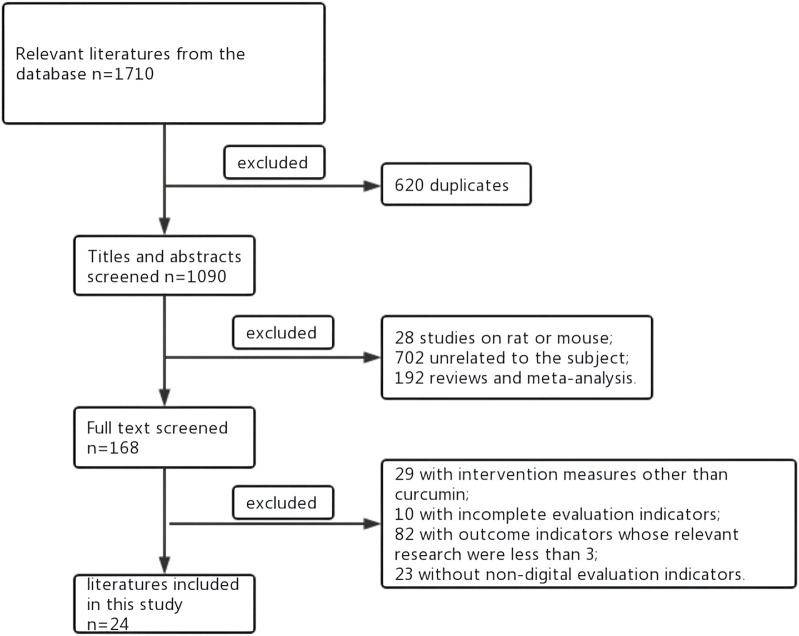
Document retrieval flow chart.

The literature review flow chart is shown in Figure 1. As shown in Figure 1, a total of 1,710 studies were retrieved from the databases. Of these, 620 were repeated studies that were then excluded, and 1,090 studies remained. Among those that remained, 702 studies were unrelated to the subject, 192 were reviews and meta-analyses, 28 were non-rat or non-mouse studies. There were 29 studies that involved intervention measures other than curcumin, 23 that involved non-digital evaluation indicators, and 10 that had incomplete evaluation indicators. There were 82 studies that involved outcome indicators whose relevant research were less than 3. Therefore, 24 studies were included in the final meta-analysis. The study ID, subjects, mode of administration, and other characteristics are shown in Table 1. In the included literature, if there were three groups in the article, they were sham operation group, model group and curcumin. If there were five groups in the article, it was the sham operation group, the model group and three different doses of Curcumin.

**TABLE 1:** Research ID, research subject, administration method and other research characteristics of included literatures.

Study ID	Study subjects (model group/curcumin group)	Modeling method	Administration method	Dosage (mg/kg)	Outcome indexes
HJ Wu 2021 (HJ W et al. (2021)	Rats (10/10)	Ligation LAD	Intraperitoneal injection	100	CK-MB, LDH, MDA, SOD
H Guo 2021 Guo et al. (2021)	Mice (10/10)	Ligation LAD	Gavage	500	CK-MB, LDH
JK Cui 2020 Cui et al. (2020)	Rats (15/15)	Ligation LAD	Intraperitoneal injection	30	CK-MB, LDH, SOD, MDA, myocardial infarction area
PM Boarescu 2019 (Boarescu et al. (2019a)	Rats (7/7)	Isoproterenol induction	Gavage	200	CK, LDH, MDA, SBP, DBP, HR
H Cheng 2005 Cheng and Liu, (2005)	Rats (20/20)	Ligation LAD	Sublingual vein	40	CK, LDH, myocardial infarction area
M Rahnavard 2019 Mehdi et al. (2019)	Rats (6/6)	Isoproterenol induction	Gavage	50	CK, LDH, SOD, MDA
Y Sunagawa 2012 Sunagawa et al. (2012)	Rats (3/5)	Ligation LAD	Gavage	0.5	SBP, DBP, LVEDD, heart rate, LVFS
Y Sunagawa 2011 Sunagawa et al. (2011)	Rats (8/9)	Ligation LAD	Gavage	50	SBP, DBP, myocardial infarction area, heart rate, LV/BW
Y Sunagawa 2014 Sunagawa et al. (2014)	Rats (11/11)	Ligation LAD	Gavage	50	SBP, DBP, myocardial infarction area, heart rate, LVFS, LV/BW
YS Kim 2012 Kim et al. (2012)	Rats (7/8)	Ligation LAD	Gavage	300	SBP, DBP, LVEDD, LVESD, heart rate, LVFS, LVEDP
ZF He 2015 He et al. (2015)	Rats (15/15)	Ligation LAD	Intraperitoneal injection	150	LVEDP
LL Chen 2014 Chen, (2014)	Mice (5/5)	Ligation LAD	Intraperitoneal injection	100	Apoptosis index, LVEDD, LVESD, LVFE, LVFS
J Kong 2012 Kong, (2012)	Rats (7/7)	Ligation LAD	Gavage	100	Apoptosis index
Y Wang 2008 Wang, (2008)	Rats (10/10)	Ligation LAD	Intraperitoneal injection	200	Apoptosis index, +dp/dtmax, -dp/dtmax
JZ Gao 2011 Gao et al. (2011)	Rats (10/10)	Ligation LAD	Intraperitoneal injection	200	Apoptosis index
SX Yan 2021 (Shaoxi et al. (2021)	Mice (7/9)	Ligation LAD	Intraperitoneal injection	100	LVEDD, LVESD, heart rate, LVFE, LVFS
JH Xu 2015 Xu and Yang, (2015)	Rats (8/8)	Ligation LAD	Free drink	-	LVEDD, LVESD, LVFE
HH Geng 2016 Geng, (2016)	Mice (8/8)	Ligation LAD	Gavage	50	Myocardial infarction area
HP Gu 2016 Gu et al. (2016)	Mice (10/10)	Ligation LAD	Intraperitoneal injection	100	Myocardial infarction area
CJ Gao 2015 Gao et al. (2015)	Rats (8/9)	Ligation LAD	Intraperitoneal injection	50	Myocardial infarction area, LVEDP, LV/BW, +dp/dtmax, -dp/dtmax
CH Liu 2015 Liu et al. (2015)	Rats (8/12)	Ligation LAD	Intraperitoneal injection	100	Heart rate, LVFE, LVFS
DS Hong 2009 Hong et al. (2009)	Rats (12/10)	Ligation LAD	Gavage	75	LVEDP
LL Chen 2015 (Chen et al. (2015)	Rats (8/8)	Ligation LAD	Caudal vein	150	LVEDP, +dp/dtmax, -dp/dtmax
HJ Liu 2018 Liu, (2018)	Rats (10/10)	Ligation LAD	Gavage	30	+dp/dtmax, -dp/dtmax

We assessed the quality of the included literature through syrcle’s bias risk tool, as shown in Table 2. Only 12.5% (*n* = 3) described the random sequence generation method like completely random design, Simple random sampling method and random block method (Hong et al., 2009; Liu et al., 2015; Gu et al., 2016); 25% (*n* = 6) reported random outcome assessments (Wang, 2008; Kong, 2012; Chen, 2014; He et al., 2015; Liu et al., 2015; Mehdi et al., 2019). Additionally, three studies that mentioned blinding only blinded echocardiographic studies, not other included parameters like MI area, HR, SBP, and DBP (Sunagawa et al., 2011; Sunagawa et al., 2012; Sunagawa et al., 2014). None of the studies reported details of baseline characteristics of animals, details of methods for allocation concealment, random housing, and blinding (high risk of bias = 100%), all studies had no incomplete results and selective results. None of the studies had a published pre-specified protocol.

**TABLE 2:** SYRCLE’s RoB tool for each experimental animal studies.

	Random sequence generation	Baseline characteristics	Allocation concealment	Random housing	Blinding (study team)	Random outcome assessment	Blinding (outcome assessors)	Incomplete outcome data	Selective outcome reporting
	Selection Bias			Performance Bias		Detection Bias		Attrition Bias	Reporting Bias
HJ Wu 2021 HJ W et al. (2021)	-	-	-	-	-	N	-	?	?
H Guo 2021 Guo et al. (2021)	-	-	-	-	-	N	-	?	?
JK Cui 2020 Cui et al. (2020)	-	-	-	-	-	N	-	?	?
PM Boarescu 2019 Boarescu et al. (2019a)	-	-	-	-	-	?	-	?	?
H Cheng 2005 Cheng and Liu, (2005)	-	-	-	-	-	-	-	?	?
M Rahnavard 2019 Mehdi et al. (2019)	-	-	-	-	-	+	-	?	?
Y Sunagawa 2012 Sunagawa et al. (2012)	-	-	-	-	-	N	-	?	?
Y Sunagawa 2011 Sunagawa et al. (2011)	-	-	-	-	-	N	-	?	?
Y Sunagawa 2014 Sunagawa et al. (2014)	-	-	-	-	-	N	-	?	?
YS Kim 2012 Kim et al. (2012)	-	-	-	-	-	?	-	?	?
ZF He 2015 (He et al. (2015)	-	-	-	-	-	+	-	?	?
LL Chen 2014 Chen, (2014)	-	-	-	-	-	+	-	?	?
J Kong 2012 Kong, (2012)	-	-	-	-	-	+	-	?	?
Y Wang 2008 Wang, (2008)	-	-	-	-	-	+	-	?	?
JZ Gao 2011 Gao et al. (2011)	-	-	-	-	-	?	-	?	?
SX Yan 2021 (Shaoxi et al., 2021)	-	-	-	-	-	?	-	?	?
JH Xu 2015 Xu and Yang, (2015)	-	-	-	-	-	?	-	?	?
HH Geng 2016 Geng, (2016)	-	-	-	-	-	?	-	?	?
HP Gu 2016 Gu et al. (2016)	+	-	-	-	-	?	-	?	?
CJ Gao 2015 (Gao et al., 2015)	-	-	-	-	-	?	-	?	?
CH Liu 2015 Liu et al. (2015)	+	-	-	-	-	+	-	?	?
DS Hong 2009 Hong et al. (2009)	+	-	-	-	-	?	-	?	?
LL Chen 2015 Chen et al. (2015)	-	-	-	-	-	?	-	?	?
HJ Liu 2018 Liu, (2018)	-	-	-	-	-	?	-	?	?

Note:+: low risk of bias -: high risk of bias ? unclear risk of bias N: not applicable.

The effects of curcumin on rats or mice centered on three aspects: blood biochemistry, cardiac tissue structure, and hemodynamics. In terms of blood biochemistry, the indicators measured were creatine kinase (CK), creatine kinase isoenzyme (CK-MB), malondialdehyde (MDA), and biochemical blood indicators, such as lactate dehydrogenase (LDH) and superoxide dismutase (SOD). The hemodynamic indexes measured included systolic blood pressure (SBP), diastolic blood pressure (DBP), heart rate (HR), left ventricular end-diastolic pressure (LVEDP), left ventricular ejection fraction (LVEF), left ventricular fractional shortening (LVFS), maximum rate of left ventricular pressure rise (+dp/dtmax), and maximum rate of left ventricular pressure decline (–dp/dtmax). The cardiac tissue structure examined included the left ventricular weight to body weight ratio (LVW/BW), apoptosis index (number of apoptotic nuclei/[number of apoptotic nuclei + number of normal nuclei] × 100%), left ventricular end-diastolic dimension (LVEDD), left ventricular end-systolic diameter (LVESD), and myocardial infarction area.

### The effect of curcumin on blood biochemistry in rats or mice with myocardial infarction

The present study sorted and merged related studies on the effects of curcumin on CK, CK-MB, MDA, and LDH levels in rats or mouse models of myocardial infarction. The results showed that curcumin could reduce CK, CK-MB, MDA, and LDH levels in myocardial infarction models but had no significant impact on SOD levels in myocardial infarction models.

**FIGURE 2:**
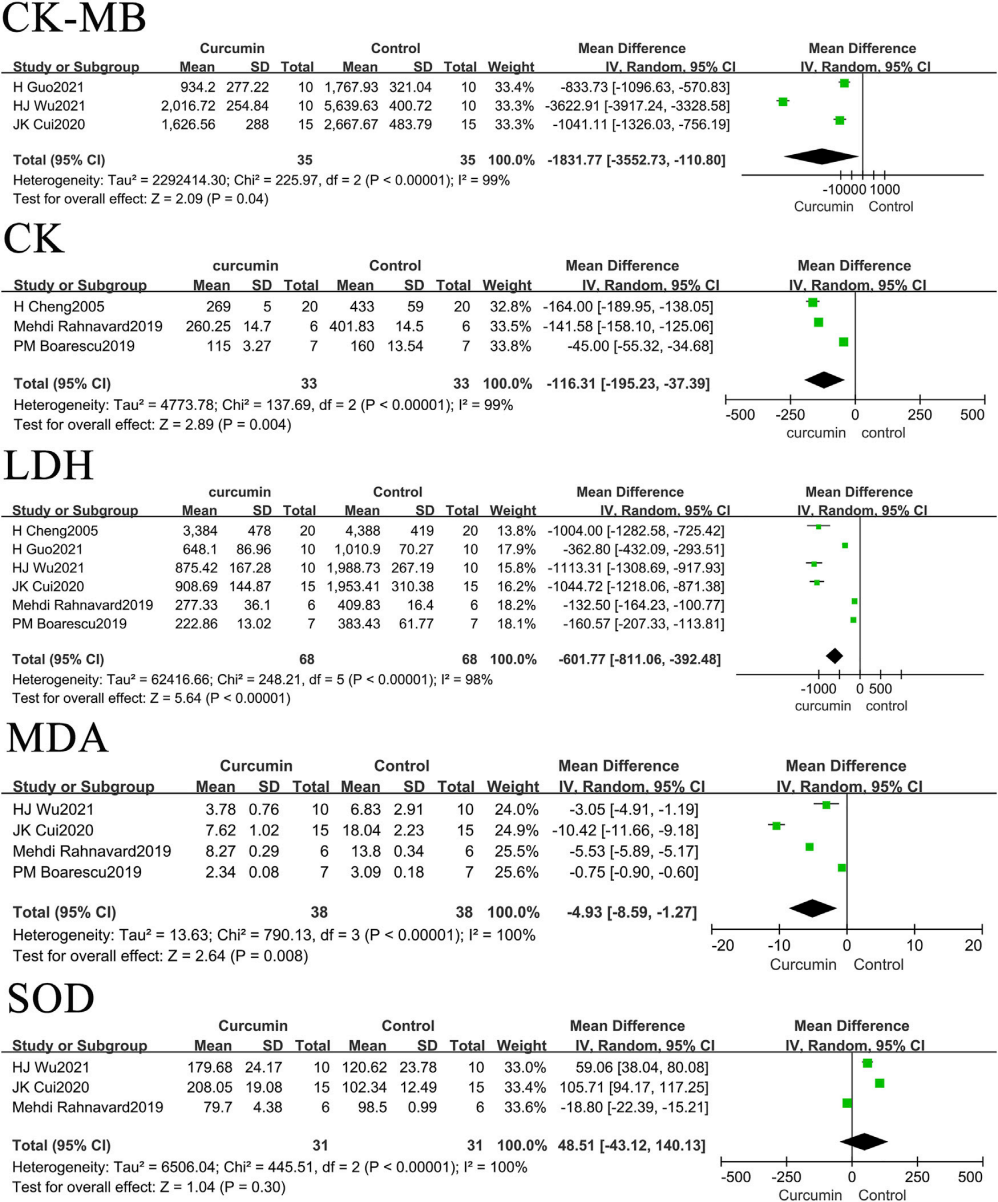
Forest plot of blood biochemical related indicators: CK-MB, CK, LDH, MDA, SOD.

The results also found that the use of curcumin can reduce the levels of CK-MB by 1,831.77 U/L (*p* = 0.04), CK by 116.31 U/L (*p* = 0.004), LDH by 601.77 U/L (*p* < 0.00001), and MDA by 4.93 nmol/L (*p* = 0.008) in rats or mice with myocardial infarction. The forest map is presented in Figure 2.

### The effect of curcumin on hemodynamic indexes in rats or mice with myocardial infarction

**FIGURE 3:**
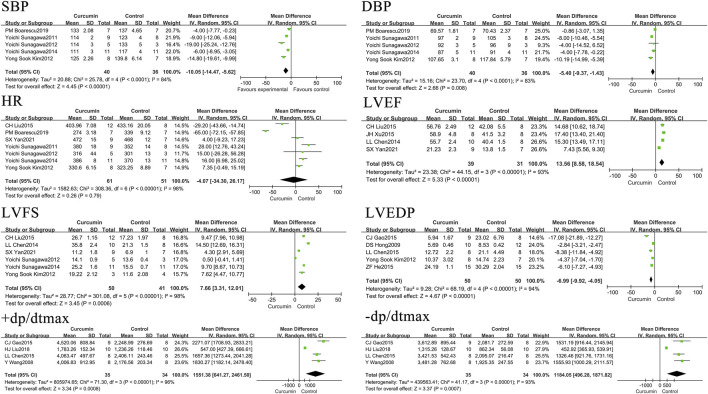
Forest plot of hemodynamic related indicators: SBP, DBP, HR, LVEF, LVFS, LVEDP, +dp/dtmax, - dp/dtmax.

In rat or mouse myocardial infarction models, curcumin was found to reduce SBP by 10.05 mmHg (*p* < 0.00001) and DBP by 5.4 mmHg (*p* = 0.008). It was also found to increase LVEF by 13.56% (*p* < 0.00001), LVFS by 7.66% (*p* = 0.0006), LVEDP by 6.99 mmHg (*p* < 0.00001), +dp/dtmax by 1551.38 mmHg/s (*p* = 0.0008), and–dp/dtmax by 1184.05 mmHg/s (*p* = 0.0007). It had no significant impact on the heart rate in rats or mice models of myocardial infarction. The forest map is presented in Figure 3.

### The effect of curcumin on cardiac tissue and structure in rats or mice with myocardial infarction

**FIGURE 4:**
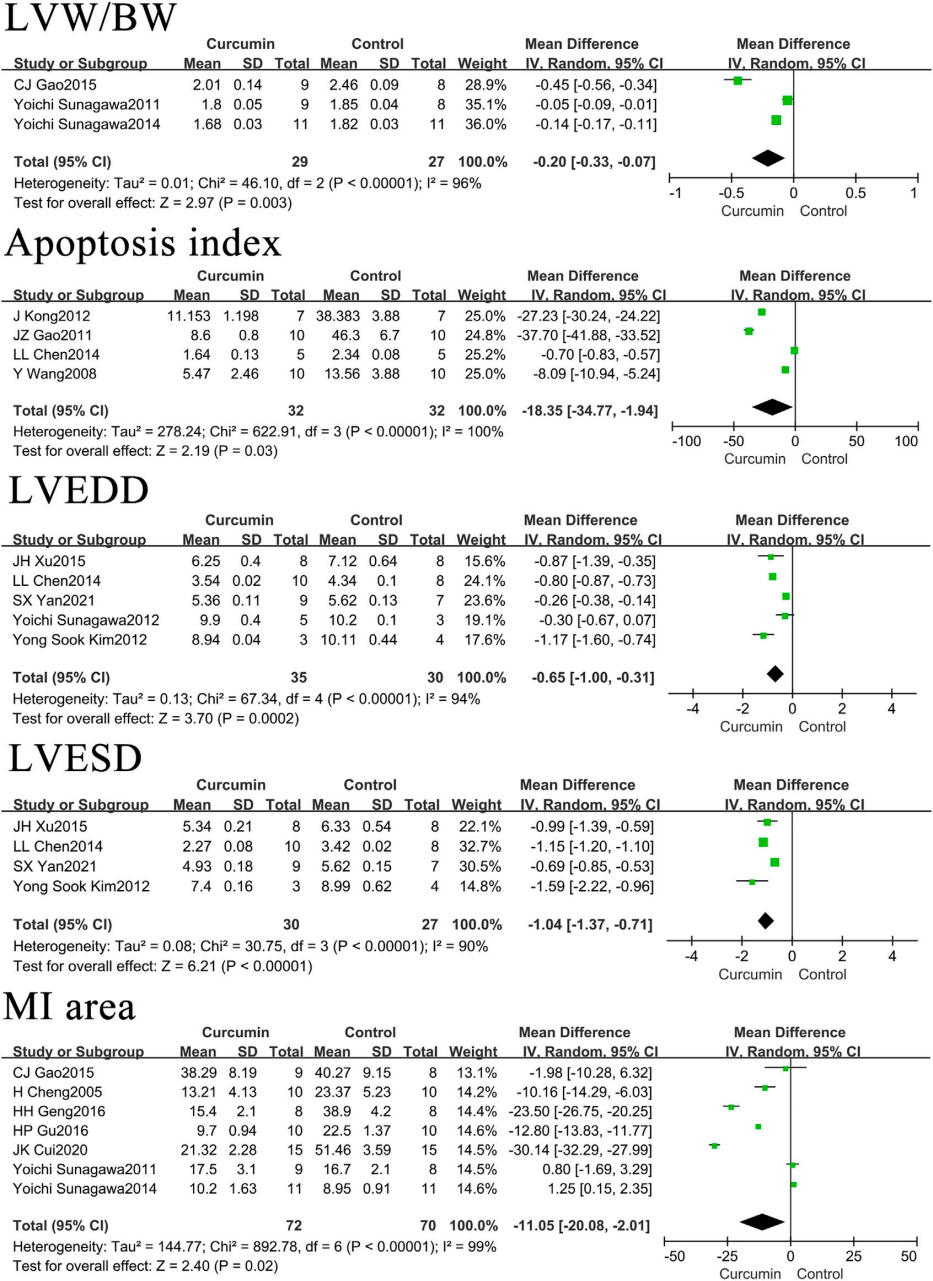
Forest plot of cardiac tissue structure related indicators: LVW/BW, Apoptosis index, LVEDD, LVESD, MI area.

Curcumin was found to reduce the LVW/BW by 0.2 (*p* = 0.003), the apoptosis index by 18.35% (*p* = 0.03), LVEDD by 0.65 mm (*p* = 0.0002), LVESD by 1.04 mm (*p* < 0.00001), and myocardial infarction area by 11.05% (*p* = 0.02). The forest map is presented in Figure 4.

### Sub study of rats and mice

In order to study the effects of different animal species on the effects of curcumin on myocardial infarction, we classified the included literatures according to rats and mice.

**FIGURE 5:**
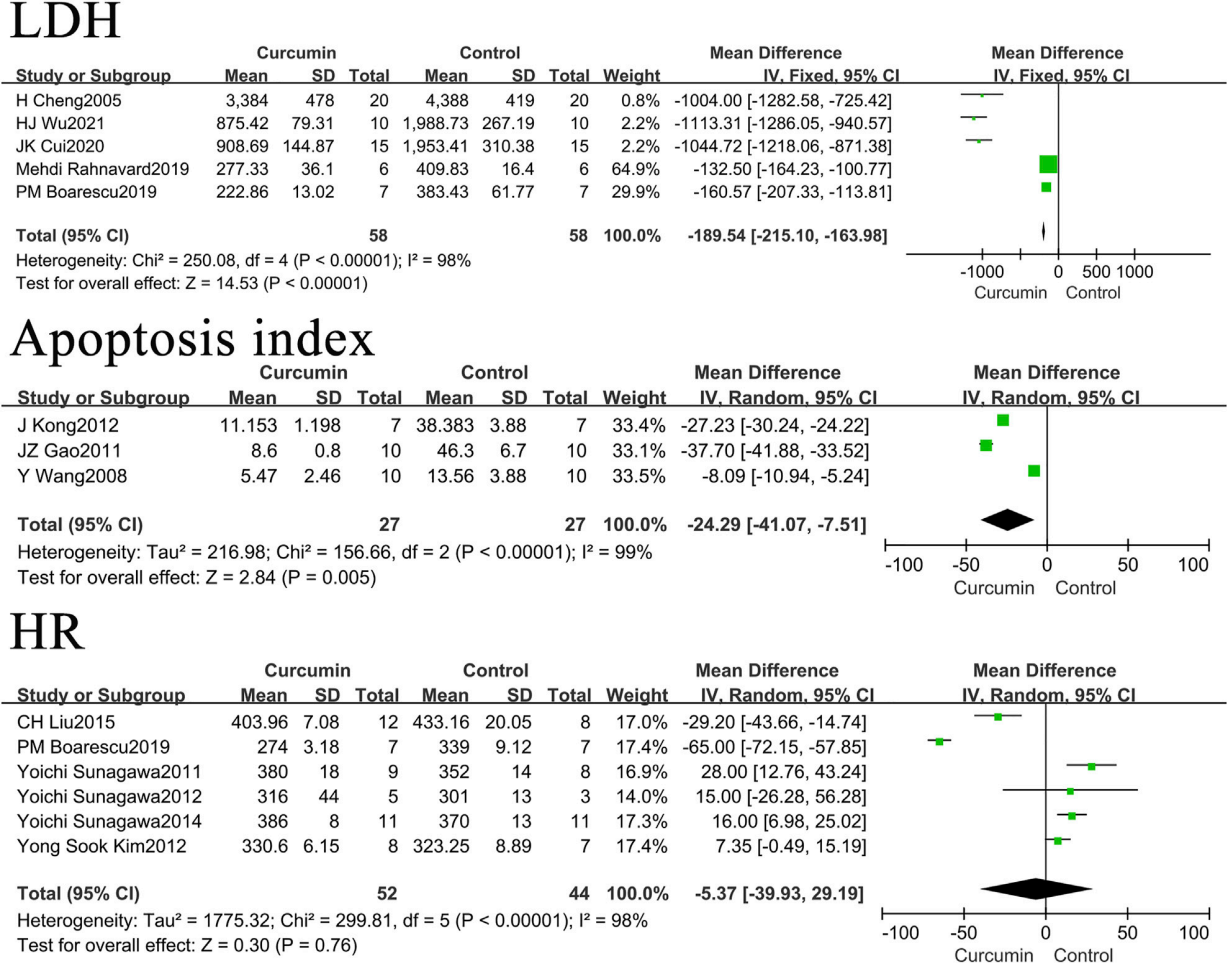
Forest plot of LDH, heart rate and apoptosis index of rats.

Among the included research literatures on LDH, one is about the mouse myocardial infarction model, and the heart rate and apoptosis index are the same. We removed this study to observe the impact on the research results, as shown in Figure 5. The results found that the use of curcumin can reduce the levels of LDH by -189.54U/L (*p* < 0.00001), the apoptosis index by 24.29% (*p* = 0.005) in rats or mice with myocardial infarction. It had no significant impact on the heart rate in rats’ models of myocardial infarction. These results are not very different from those before the removal of mice.

**FIGURE 6:**
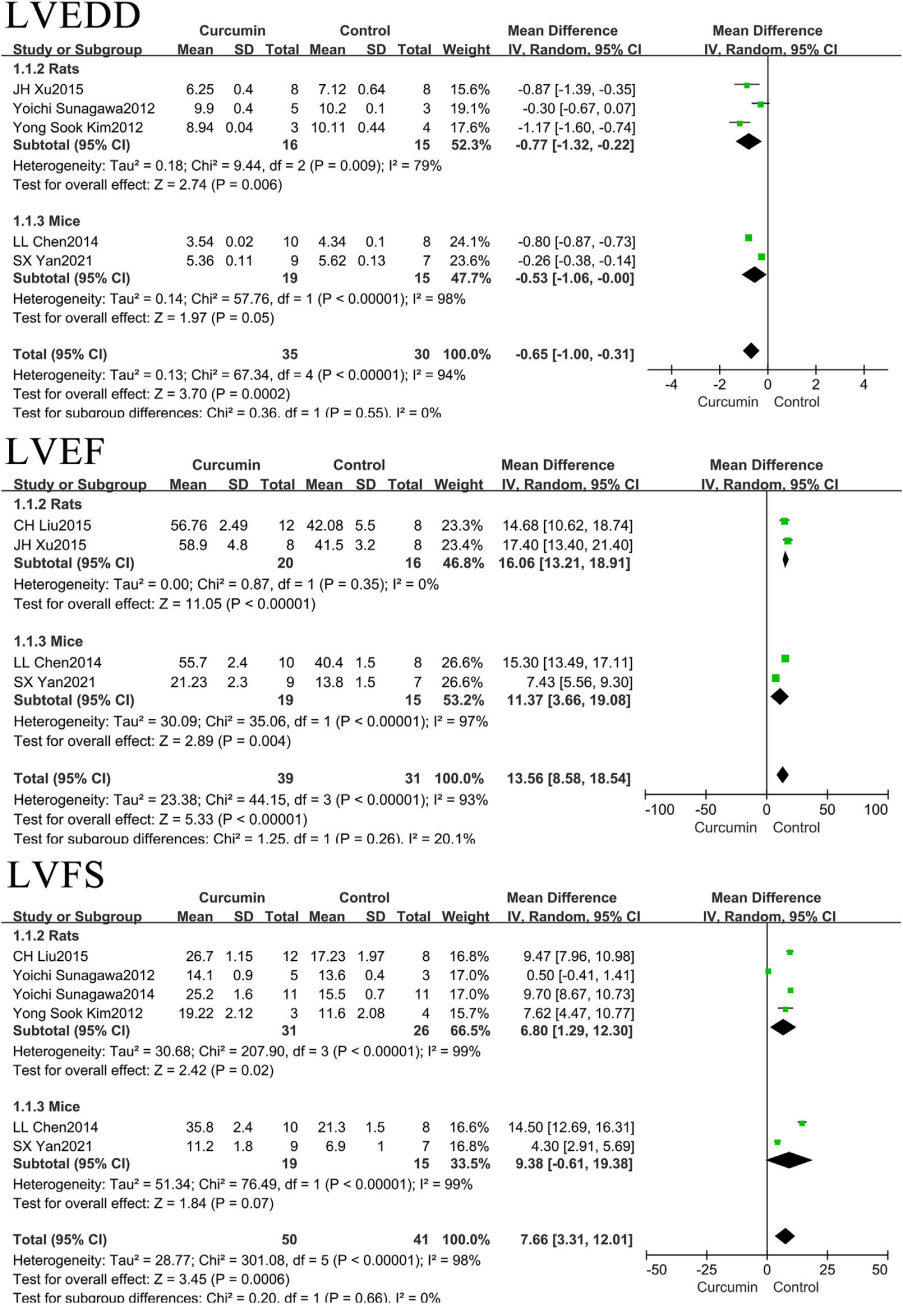
Forest plot of LVEDD, LVEF and LVFS in rats and mice, respectively.

In our relevant research literature, rats and mice were included in the literature of LVEDD, LVEF and LVFS. We classified the rats and mice, as shown in Figure 6. The results found that the use of curcumin can reduce LVEDD by 0.77 mm (*p* = 0.006) in rats with myocardial infarction, LVEDD by 0.53 mm (*p* = 0.05) in mice with myocardial infarction. Curcumin was found to increase LVEF by 16.06% (*p* < 0.00001) in rats with myocardial infarction, LVEF by 11.37% (*p* = 0.004) in mice with myocardial infarction. Curcumin was found to increase LVFS by 6.80% (*p* = 0.02) in rats with myocardial infarction, LVFS by 7.66% (*p* = 0.0006) in mice with myocardial infarction.

## Discussion

In this meta-analysis, we found that curcumin could reduce CK-MB, CK, LDH, and MDA levels. Also, it could lower SBP, DBP, LVEDP, LVW/BW, apoptosis index, LVEDD, LVESD, and myocardial infarction area and increase LVEF, LVFS, +dp/dtmax, and–dp/dtmax. However, it had no significant impact on the heart rate and the levels of SOD in the models.

The levels of CK, CK-MB, and LDH are commonly used markers of myocardial injury, as their changes can reflect the degree of myocardial injury. In myocardial infarction models, curcumin reduces CK, CK-MB, and LDH levels and alleviates myocardial injury. Inflammatory reaction and oxidative stress are the initiating and promoting factors of myocardial infarction, existing throughout the process of myocardial infarction. A by-product of lipid peroxidation in oxidative stress is MDA, changes in which can reflect the degree of oxidative stress. Curcumin reduces the MDA concentration in myocardial infarction models, indirectly confirming the antioxidant effect of curcumin.

Curcumin has also been found to reduce SBP, DBP, and LVEDP. Blood pressure has an important impact on the prognosis of acute myocardial infarction. However, in human epidemiological studies, there has been debate about the reasonable control range of blood pressure. An international cohort study observing data from 22,672 patients with coronary atherosclerotic heart disease demonstrated that when SBP was controlled between 120 and 140 mmHg (1 mmHg = 0.1333 kPa), the incidence of cardiovascular events was the lowest. However, an SBP <120 mmHg was associated with an increased risk of cardiovascular death and all-cause death, and a mean follow-up of 5 years revealed that an SBP ≥140 mmHg or <120 mmHg and a DBP ≥80 mmHg or <70 mmHg were associated with an increased risk of cardiovascular events and showed a J-curve relationship (Selvaraj et al., 2016).

Curcumin reduces indicator numbers of cardiac structure, including LVW/BW, LVEDD, and LVESD, reduces left ventricular dilatation, alleviates myocardial hypertrophy and remodeling, increases cardiac systolic function indicators, including LVFS, LVEF, +dp/dtmax, and–dp/dtmax, and improves the diastolic and systolic capacity of the ventricle.

Myocardial apoptosis is the main form of myocardial cell loss in the early stages of myocardial ischemia (Wang et al., 2018). During myocardial ischemia, the apoptosis mechanism is first initiated. With time and dependent on the aggravation of the degree of ischemia, the cells in the ischemic center then progress to necrosis. Cardiomyocytes around the ischemia are then in apoptosis, a state of reversible damage. This is why, in acute myocardial infarction, apoptotic cells appear in the center and periphery of the infarction, and more apoptotic cells are found in the peripheral area of the infarction (Jo et al., 2020; Zhou et al., 2020; Sheng et al., 2021). Curcumin reduces the myocardial infarction area and apoptosis index in rat or mouse myocardial infarction models and alleviates myocardial necrosis and apoptosis.

In some of the included studies, the dosage of curcumin was divided into several levels. The results of almost all such articles showed that the dosage of curcumin showed a dose effect with different dosage, and the effect of curcumin increased with the increase of dosage. In our study, the research results of the maximum dosage included in the study were selected. Because the dose distribution of the included studies was quite different, it was not possible to classify the doses for the time being.

## Conclusion

Curcumin alleviates myocardial injury and oxidative stress in myocardial infarction rodent models in terms of blood biochemistry indicators, improves the diastolic and systolic capacity of the ventricle in terms of hemodynamics, and reduces the necrosis and apoptosis of cardiomyocytes in terms of tissue structure. These results show the valuable effects of curcumin on myocardial infarction in the clinic. However, the methodological quality of the studies was low. Additional research is warranted focusing on rigour of assessment, intensity of interventions delivered and methodological limitations.

## Data Availability

The raw data supporting the conclusion of this article will be made available by the authors, without undue reservation.
